# Hypoxia-inducible factor-1*α* and -2*α* are expressed in most rectal cancers but only hypoxia-inducible factor-1*α* is associated with prognosis

**DOI:** 10.1038/sj.bjc.6605026

**Published:** 2009-05-12

**Authors:** S Rasheed, A L Harris, P P Tekkis, H Turley, A Silver, P J McDonald, I C Talbot, R Glynne-Jones, J M A Northover, T Guenther

**Affiliations:** 1 Department of Surgery, St Mark's Hospital, Harrow, Middlesex, UK; 2 Weatherall institute of Molecular Medicine, John Radcliffe Hospital, Oxford, UK; 3 Department of Surgical Oncology and Technology, Royal Marsden and Chelsea and Westminster Hospitals, Imperial College, London, UK; 4 Colorectal Cancer Genetics, St Bartholomew's and The London Hospital, Institute of Cell and Molecular Sciences, Queen Mary's University of London, London, UK; 5 Academic Department of Cellular Pathology, St Mark's Hospital, Harrow, Middlesex HA1 3UJ, UK; 6 Department of Oncology, Mount Vernon Hospital, Northwood, Middlesex, UK

**Keywords:** rectal cancer, angiogenesis, hypoxia, HIF-1*α* and HIF-2*α*, microvessel density

## Abstract

The hypoxia-mediated response of tumours is a major determining factor in growth and metastasis. Understanding tumour biology under hypoxic conditions is crucial for the development of antiangiogenic therapy. Using one of the largest cohorts of rectal adenocarcinomas to date, this study investigated hypoxia-inducible factor-1*α* (HIF-1*α*) and HIF-2*α* protein expression in relation to rectal cancer recurrence and cancer-specific survival. Patients (*n*=90) who had undergone surgery for rectal adenocarcinoma, with no prior neoadjuvant therapy or metastatic disease, and for whom adequate follow-up data were available were selected. Microvessel density (MVD), HIF-1*α* and HIF-2*α* expressions were assessed immunohistologically with the CD34 antibody for vessel identification and the NB100-131B and NB100-132D3 antibodies for HIF-1*α* and HIF-2*α*, respectively. In a multifactorial analysis, results were correlated with tumour stage, recurrence rate and long-term survival. Microvessel density was higher across T and N stages (*P*<0.001) and associated with poor survival (hazard ratio (HR)=8.7, *P*<0.005) and decreased disease-free survival (HR=4.7, *P*<0.005). hypoxia-inducible factor-1*α* and -2*α* were expressed in >50% of rectal cancers (HIF-1*α*, 54%, 48/90; HIF-2*α*, 64%, 58/90). HIF-1*α* positivity was associated with both TNM stage (*P*<0.05) and vascular invasion (*P*<0.005). In contrast, no associations were shown between HIF-2*α* expression and any pathological features, and HIF-1*α* positivity had no effect on outcome. The study showed an independent association between HIF-1*α* expression and advanced TNM stage with poor outcome. Our results indicate that HIF-1*α*, but not HIF-2*α*, might be used as a marker of prognosis, in addition to methods currently used, to enhance patient management.

The UK incidence of colorectal carcinoma is approximately 34 000 of which a third are rectal cancers (Kmietowicz, 2004). In the United States, colorectal cancer is the second most common cancer in women and the third commonest in men with over 148 000 new cases each year (American Cancer Society, 2006). After potentially curative surgical resection, local recurrence rates of rectal cancer have been reported from 4 to 32% (Sagar and Pemberton, 1996) with overall 5 year survival of less than 40% (Lindmark *et al*, 1994; Dahlberg *et al*, 1998).

Hypoxia is one of the key stimuli for the release of angiogenic factors necessary for angiogenesis and tumour growth. Tumours outgrow their local blood supply resulting in a hypoxic microenvironment. Although lesions 1–2 mm in diameter receive nutrients by cellular diffusion, an increase in tumour size beyond this requires rapid adaptation to hypoxia to prevent cessation of growth and necrosis (Pugh and Ratcliffe, 2003). A component of this adaptation, increased microvessel density (MVD), has been shown in polyps and colorectal cancers (Bossi *et al*, 1995) and linked to increasing transmural tumour extension (Choi *et al*, 1998; Rasheed *et al*, 2009). High colorectal cancer MVD has also been associated with an increased incidence of haematogenous metastases and poor prognosis (Tomisaki *et al*, 1996; Gulubova and Vlaykova, 2007; Rasheed *et al*, 2008).

Hypoxia-inducible factor (HIF) is a heterodimeric basic helix-loop-helix transcription factor involved in the regulation of cellular adaptation to hypoxia by upregulating genes directly responsible for angiogenesis. HIF consists of an *α-* and *β*-subunit (HIF-*α* and HIF-*β*/ARNT; Conway *et al*, 2001), which can be further subdivided into distinct isoforms, including HIF-1, HIF-2 and HIF-3. The first two of these exhibit conserved amino-acid sequences, but different mRNA expression patterns (Conway *et al*, 2001; Aprelikova *et al*, 2004). The HIF-*β*/ARNT subunit is constitutively expressed and involved in a number of non-hypoxia-related processes, whereas the *α*-subunit is the hypoxia-regulating component (Carmeliet *et al*, 1998; Talks *et al*, 2000). Both HIF-1*α* and HIF-2*α* are subject to post-transcriptional regulation mediated by the von Hippel–Lindau protein (VHL), but only HIF-1*α* regulates the induction of the glycolytic enzymes essential for cell proliferation and survival under hypoxic stress (Aprelikova *et al*, 2004). Under physiological normoxic conditions, HIF-*α* is propyl hydroxylated after activation of VHL and then prepared for degradation through mitochondrial ubiquitination. However, under hypoxic conditions, this degradation cannot take place due to a reduction in propyl hydroxylation, which leads to HIF-1*α* accumulation; levels of HIF-1*α* correlate with the oxygen status of the cell (Iyer *et al*, 1998; Blancher *et al*, 2000). HIF-*α* upregulation causes activation of target genes and expression of various growth factors, including VEGF, which induce endothelial cell proliferation and migration resulting in new vessel growth (Ratcliffe *et al*, 1997; Talks *et al*, 2000; Giatromanolaki and Harris, 2001).

Patients with mutations in the VHL tumour suppressor gene develop highly vascular tumours, including renal cell carcinomas, phaeochromocytomas and retinal haemangioblastomas (Semenza, 2001; Wykoff *et al*, 2001). Hypoxia-inducible factor has been shown to play a major role in the angiogenesis and growth of various tumours, including breast (Leek *et al*, 2002), bladder (Palit *et al*, 2005), renal (Klatte *et al*, 2007), pancreatic (Shibaji *et al*, 2003) and cervical (Birner *et al*, 2000) cancers. Indeed, overexpression of, in particular, HIF-1*α*, has been correlated with unfavourable prognosis in a number of malignancies (Theodoropoulos *et al*, 2005; Maynard and Ohh, 2007; Trastour *et al*, 2007).

In colorectal cancer, Jiang *et al* (2003a, 2003b) showed that HIF-1*α* mRNA was present in a significant number of colorectal adenoma and carcinoma specimens. They also found an increase in HIF-1*α* expression concordant with more advanced Dukes’ stage. Kuwai *et al* (2003) associated HIF-1*α* expression with tumour invasion, venous invasion, liver metastasis and vascular endothelial growth factor (VEGF) expression. Similarly, Lu *et al* (2006) found a strong association between HIF-1*α* expression, high VEGF expression, nodal metastasis and Dukes’ stage in rectal cancer and, in contrast to Kuwai *et al* (2003) showed an overall reduction in survival in patients with high HIF-1*α* expression. These findings were supported by an investigation in patients with locally advanced rectal cancer where an association was shown between HIF-1*α* expression, lymph node metastasis and poor outcome. Antiangiogenic drugs have been shown to be effective both experimentally and in clinical trials in various malignancies, including colorectal cancer. The monoclonal IgG_1_ antibody, Bevacizumab (Avastin), which binds VEGF preventing receptor binding, has been evaluated in the first- and second-line treatment in patients with colorectal cancer (Willett *et al*, 2004, 2006; Chua and Cunningham, 2006; Rasheed *et al*, 2008). Interestingly, novel antiangiogenic compounds have been developed that decrease HIF-1*α* and other HIFs (Mackay *et al*, 2005; Zhu *et al*, 2007; Singh *et al*, 2008).

We are evaluating whether detection of hypoxic factor expression in rectal cancer can be used to identify patient subgroups at increased risk of recurrences and poorer outcome. Recognising positive factor expression will identify patients most likely to benefit from specific antiangiogenic therapies, including HIF inhibitors. In this study, we investigate one of the largest cohorts of patients to date with rectal adenocarcinoma across all stages for HIF-1*α* and HIF-2*α* expressions and have appraised associations between expression and a number of clinicopathological variables.

## Materials and methods

Patients with rectal adenocarcinoma operated on between 1991 and 1997 at St Mark's Hospital were selected for this study provided they had curable, local disease before surgery. Those with metastatic disease and/or had received neoadjuvant chemoradiotherapy were excluded. Complete follow-up data and sufficient paraffin-embedded tissue to stain with antibodies for HIF-1*α*, HIF-2*α* and CD34 (for MVD) were available in 90 cases. Normal proximal adjacent bowel obtained from 25 randomly selected patients was used as internal control tissue.

**Figure 1:**
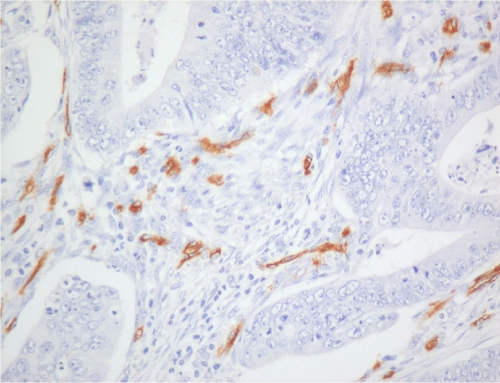
Microvessels stained with CD34 monoclonal antibody within rectal adenocarcinoma showing high vessel density ( × 200).

**Figure 2:**
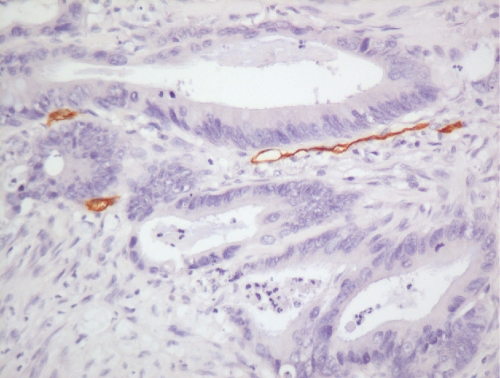
Microvessels stained with CD34 monoclonal antibody within rectal adenocarcinoma showing low vessel density ( × 200).

Tissue sections (4 *μ*m) of archival paraffin-embedded block specimens were mounted onto slides. The antibodies used were for identification of CD34 blood vessels (Novus Biologicals, LLC, Littleton, CO, USA, QBend10, dilution 1 : 100, no pre-treatment), HIF-1*α* (NB 100-131B, Novus Biologicals, dilution 1 : 500, pressure cooked for 2 min at full pressure, pre-treated with citrate buffer, pH 6.0) and HIF-2*α* (NB100-132D3, Novus Biologicals, dilution 1 : 100, pressure cooked for 2 min at full pressure, pre-treated with citrate buffer, pH 6.0; Talks *et al*, 2000; Koukourakis *et al*, 2002; Leek *et al*, 2002). Immunohistochemistry was undertaken in a DAKO Immuno Autostainer Plus (Glostrup, Denmark) with ‘Chemmate Envision’ after standard dewaxing with xylene and ethanol and pre-treatment. Microvessel density was assessed by counting vessels per power field (23-mm eye piece width) at × 200 magnification following identification of the three most vessel-dense ‘hotspots’ at × 40 magnification (Figures 1 and 2; Bossi *et al*, 1995). Vessels were counted in the three most vessel-dense areas within the central part of the tumour and at the invasive tumour front. A mean score of all areas was calculated. The mean value of 60 vessels per high power field was used as the cutsoff between high and low MVDs.

**Figure 3:**
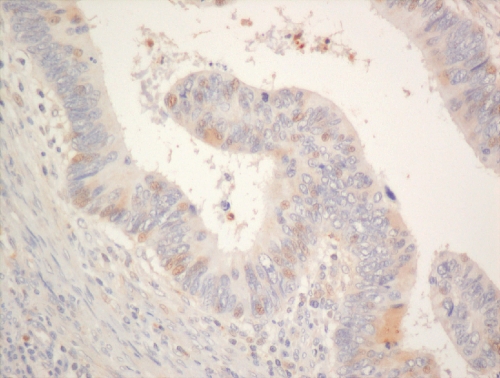
Hypoxia-inducible factor-1*α* nuclear staining in rectal adenocarcinoma ( × 200).

**Figure 4:**
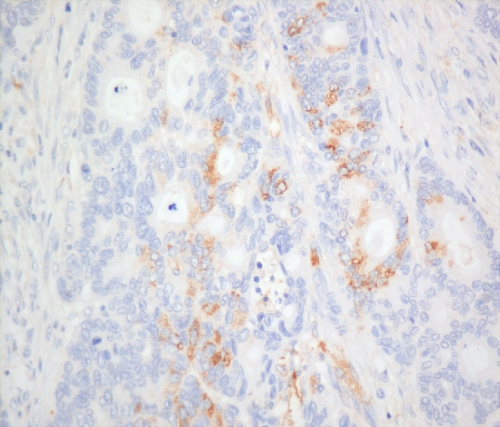
Cancer and macrophage cytoplasmic staining for HIF-2*α* ( × 200).

**Figure 5:**
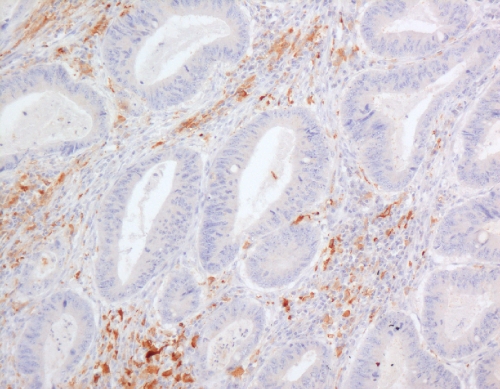
Predominant macrophage staining for HIF-2*α* ( × 100).

Hypoxia-inducible factor-1*α* and -2*α* (nuclear and cytoplasmic) stainings (Figures 3, 4 and 5) were reviewed by two experienced assessors (SR and TG) in terms of percentage of positive tumour cells. The predominant site of distribution, for example, intratumoral, invasive edge of tumour was noted. The following clinicopathological factors were analysed: age at the time of surgery; gender; surgical procedure categorised as abdominoperineal excision of rectum or anterior resection of rectum; tumour grading; staging according to the Dukes’ and tumour node metastasis (TNM) classifications; venous invasion (intramural and extramural); perineural invasion, number of regional lymph nodes and number of involved lymph nodes.

### Data sources and study approval

The study was approved by the North West London Hospitals’ Research and Development Committee and the Harrow Research and Ethics Committee.

### Statistical analysis

Statistical analysis was carried out using SPSS for Windows version 14 (SPSS Inc, Chicago, IL, USA). The Fisher's exact test or Yates continuity corrected *χ*^2^-test was used for testing relationships between categorical variables where appropriate. Kaplan–Meier survival curves were constructed, and the log-rank test was used to determine statistical differences between the groups. A Cox proportional hazard model was used to assess the effects of patient and tumour variables on survival with *P*<0.05 taken as significant. Hazard ratios (HRs) were calculated to assess the independent relationship of variables with cancer-specific and disease-free survivals.

## Results

### Patients and clinicopathogical characteristics

A total of 90 patients comprising 56 males (62%), 34 females (38%) were studied with a mean age of 59 (standard deviation, s.d.±12) years and a median follow-up of 78 months (range: 2–228 months). There was no significant difference between male and female patients in terms of mean age or follow-up. Clinicopathological characteristics are described in Table 1.

### Microvessel density differs between tumour and normal tissue, across T and N stages and is associated with poor survival

**Figure 6:**
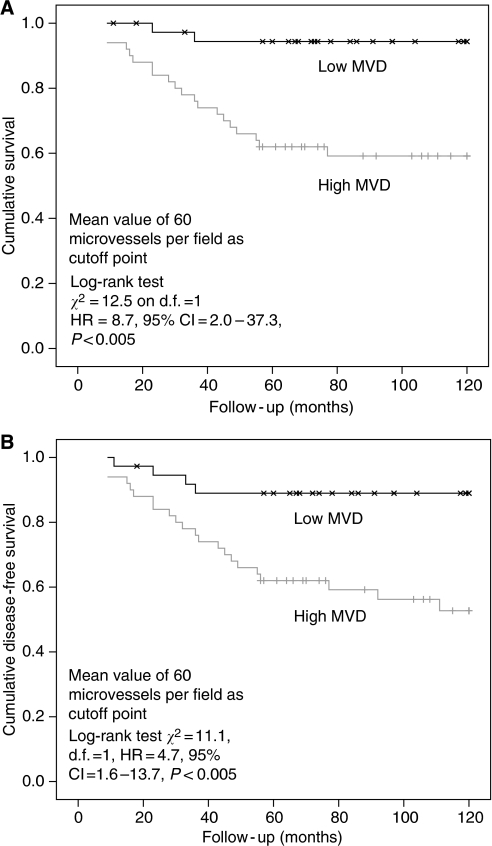
(**A**) Kaplan–Meier cancer-specific survival curve of patients grouped by high and low microvessel density (MVD). (**B**) Kaplan–Meier disease-free survival curve of patients grouped by high and low (MVD).

For the cases (*n*=90), the mean MVD was 63 vessels per high-power field (HPF; s.d.±20) and for controls (*n*=23), the MVD was 22 vessels per HPF. The MVD was significantly higher in tumour *versus* non-neoplastic mucosa (63 *vs* 22; *P*<0.01, Fisher's exact) and across the various T stages: T1=44 (s.d.±3); T2=45 (s.d.±13); T3=67 (s.d.±21) and T4=68 (s.d.±9; *post hoc*, ANOVA, *P*<0.001); and N stages: N0=56 (s.d.±16), N1=74 (s.d.±17), N2=76 (s.d.±26; *post hoc*, ANOVA, *P*<0.001). Survival analysis showed a significant difference in cancer-specific survival in the high *versus* low MVD groups divided by the median MVD of 60 vessels per HPF (Figure 6A; log-rank test *χ*^2^=12.5, d.f.=1 (degree of freedom); HR=8.7, 95% confidence interval (CI:) 2.0–37.3, *P*<0.005) and disease-free survival (Figure 6B; log-rank test *χ*^2^=11.1, d.f.=1, HR=4.7, 95% CI: 1.6–13.7, *P*<0.005).

### HIF-1*α* but not HIF-2*α* protein expression is associated with advanced pathological features and poor survival

Hypoxia-inducible factor-1*α* and -2*α* stains were present within the epithelial and stromal compartments in over half of rectal cancers (54%, 48/90 and 64%, 58/90, respectively). In contrast, HIF-1*α* stain was only present in a small fraction of control sections (8%, 2/25; HIF-1*α* staining in cases *vs* controls, *P*<0.005) and HIF-2*α* stain was not seen in any of the control sections. The following features were observed to be associated with HIF-1*α* positivity (Table 2): lymph node stage (*P*<0.02); TNM stage (*P*<0.05); and vascular invasion (*P*<0.005).

**Figure 7:**
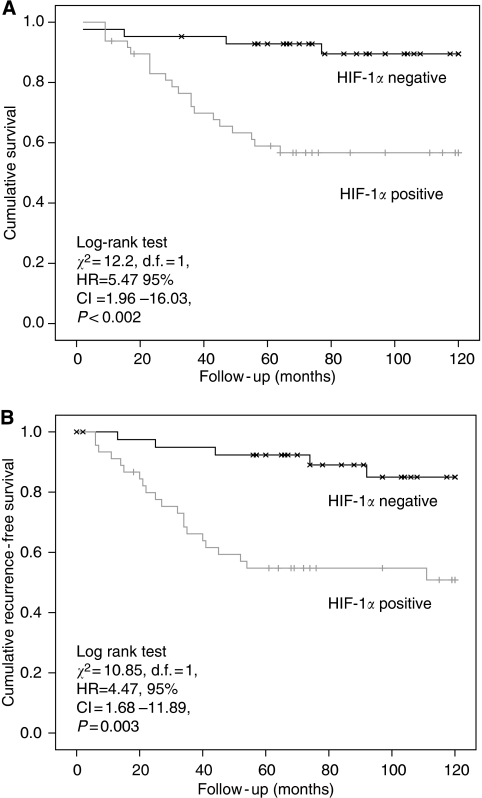
(**A**) Kaplan–Meier cancer-specific survival curve in rectal cancer patients grouped by HIF-1*α* positivity. (**B**) Kaplan–Meier disease-free survival curve in rectal cancer patients grouped by HIF-1*α* positivity.

**Figure 8:**
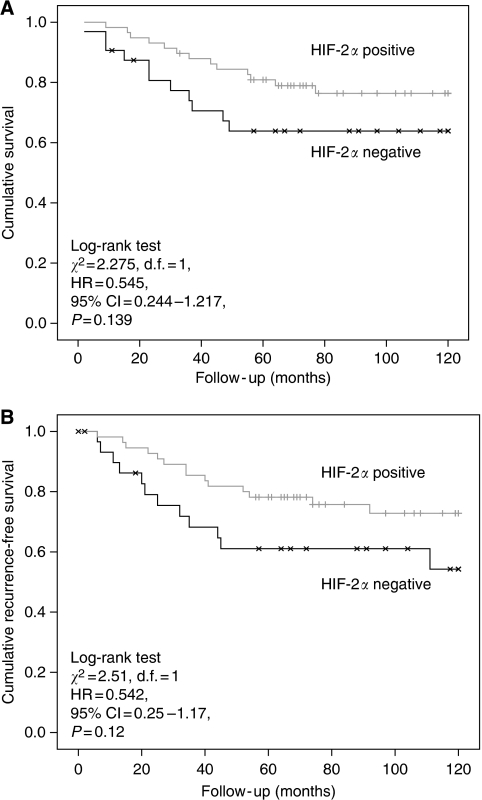
(**A**) Kaplan–Meier cancer-specific survival curve in rectal cancer patients grouped by HIF-2*α* positivity. (**B**) Kaplan–Meier recurrence-free survival curve in rectal cancer patients grouped by HIF-2*α* positivity.

The association between Dukes’ stage and HIF-1*α* positivity almost reached statistical significance (*P*=0.05), although combining Dukes’ A and B cancers together and C1 with C2 cancers resulted in a statistically significant difference in HIF-1*α* positivity between Dukes’ A/B and C (Dukes’ A/B, 44% positive *vs* 56% negative; Dukes’ C, 70% positive *vs* 30% negative; *P*<0.02). In contrast to results for HIF-1*α*, there were no observed associations between HIF-2*α* positivity and any of the pathological features under investigation (Table 3). Cox regression univariate analysis revealed a significant effect of HIF-1*α* positivity on cancer-specific survival (Figure 7A; log-rank test *χ*^2^=12.2, d.f.=1, HR=5.47 95% CI: 1.96–16.03, *P*<0.002) and disease-free survival (Figure 7B; log-rank test *χ*^2^=10.85, d.f.=1, HR=4.47, 95% CI: 1.68–11.89, *P*=0.003). On multivariate analysis, HIF-1*α* positivity retained a significant effect on cancer-specific survival independent of TNM stage and vascular invasion (HR=4.11, 95% CI=1.37–12.35, *P*=0.012; Table 4). Again, in direct contrast, there was no effect of HIF-2*α* positivity on cancer-specific survival (log-rank test *χ*^2^=2.275, d.f.=1, HR=0.545, 95% CI: 0.244–1.217, *P*=0.139; Figure 8A), although, contrary to HIF-1*α*, a trend towards decreased recurrence (log-rank test *χ*^2^=2.51, d.f.=1, HR=0.542, 95% CI=0.25–1.17, *P*=0.12) was observed for HIF-2*α* positivity (Figure 8B).

## Discussion

Our study comprises one of the largest groups of rectal cancer cases across all stages and we have determined that both HIF-1*α* and HIF-2*α* were widely expressed in rectal cancer compared with normal large bowel mucosa (controls). Over half of all cases showed HIF-1*α* expression (54%) and nearly two-thirds were found to have positive HIF-2*α* expression (64%). As discussed earlier by Talks *et al* (2000), as HIF-*α*-subunits are affected by cellular oxygenation, it is uncertain as to whether the findings in paraffin-embedded-fixed tissue accurately reflect cellular status *in vivo*. However, our observed findings do show clear differences between the two groups and, as cases and controls have been processed in a consistent manner, this does represent a genuine finding of increased hypoxic factor expression in rectal cancer cases when compared with normal rectal tissue.

We have shown an association between increasing depth of tumour invasion and MVD and have shown a relationship between increased MVD and poor prognosis, a finding consistent with earlier studies investigating colorectal cancers (Tomisaki *et al*, 1996; Choi *et al*, 1998). In a large series of 97 rectal cancer-specific patients, Chen *et al* (2004) reported that cases with a higher MVD were more likely to develop tumour recurrence or metastasis. Tomisaki *et al* (1996), in assessing an association correlation between MVD and liver metastasis, found a mean MVD of 64 in colorectal cancer patients with hepatic metastasis compared with a mean MVD of 52 in patients without metastasis (*P*=0.001). In addition, the relationship in colorectal cancer between MVD and prognosis has also been described in most studies indicating a positive correlation between high MVD and poor outcome (Vermeulen *et al*, 1999; Li *et al*, 2003). We have shown a direct relationship between increasing MVD and cancer-specific survival. In our study, an MVD of above the cutoff of 60 microvessels per HPF was a clear determinant of poor outcome. As such, we would propose that high MVD (above 60 HPF) can be used as a factor to determine surveillance and adjuvant therapy. As yet, to our knowledge, the effect of specific chemotherapeutic or antiangiogenic agents has not been assessed in a randomised controlled trial with groups determined by MVD.

Our data show an association between HIF-1*α* expression and TNM stage, nodal stage, vascular invasion and Dukes’ stage, on subdivision between node-positive and -negative cases, suggesting a direct role for HIF-1*α* in disease progression. Interestingly, we found no association between HIF-1*α* and MVD in our study, and either more numbers may be needed to show an association or it may suggest an alternative mechanism of action for this hypoxic-inducible factor. We have also shown a strong correlation between HIF-1*α* expression and cancer-specific mortality and tumour recurrence suggesting that this factor is an integral component of rectal cancer growth. Perhaps HIF is regulated by oncogenes in addition to acting through the pathway regulated by hypoxia. For example, interactions of HIF1 with *β*-catenin in colon cancer cell lines have been reported (Kaidi *et al*, 2007). Also, it is possible that as both mature and immature vessels are being assessed, this may reflect effects of other growth factors and vascular differentiation.

Curiously, although HIF-2*α* is considered to act as an oncogene, we did not find any association between the various pathological factors studied and protein expression of this gene, nor did we find an association between HIF-2*α* expression and cancer-specific survival or recurrence. We did not show a relationship between HIF-2*α* and MVD, which is also surprising. There did appear to be a trend towards a reduction in tumour recurrence with HIF-2*α* expression, although this did not reach statistical significance. Both Jiang *et al* (2003b), in colorectal cancer, and Lu *et al* (2006), in rectal cancer, showed increased positivity for HIF-1*α* expression with increasing Dukes’ stage. The latter group also found an overall reduction in survival in patients with high HIF-1*α* expression. Theodoropoulos *et al* (2006) showed a significant association between HIF-1*α* and lymph node metastasis, low rectal location and advanced tumour grade. Although HIF-1*α* expression, infiltrative tumour growth pattern, positive lymph node status and VEGF upregulation were associated with decreased disease-free and overall survival, multivariate analysis only revealed high HIF-1*α* reactivity and lymph node positivity as predictors of poor outcome. This is the more notable of the earlier rectal cancer studies due to the higher numbers, and it is interesting to note the similarities in terms of percentage of expression in these studies to our own.

It is quite likely that numerous factors act separately to cause tumour growth and spread, and pathways may interact in an as yet undefined manner. A number of these factors have been described to be hypoxia and non-hypoxia dependent, including VHL, COX-2, CA-9, CHOP and ATF4, among many others (Yoshimura *et al*, 2004; Kivela *et al*, 2005; Carracedo *et al*, 2006; Cleven *et al*, 2007).

## Figures and Tables

**Table 1:** Clinicopathological features of a cohort of rectal cancer patients

**Clinicopathological variables**	***N* (%)**
*T stage*	
T1	2 (2)
T2	16 (18)
T2	68 (76)
T3	4 (4)
T4	68 (76)
	
*N stage*	
N0	57 (63)
N1	21 (23)
N2	12 (13)
	
*Operation*	
Anterior resection	75 (83)
Abdominoperineal excision	15 (17
	
*Histogical grade*	
Well differentiated	4 (4)
Moderately differentiated	73 (81)
Poorly differentiated	13 (14)
	
*Dukes’ stage*	
Dukes’ A	17 (19)
Dukes’ B	40 (44)
Dukes’ C1	28 (31)
Dukes’ C2	5 (6)
	
*TNM stage*	
Stage I	17 (19)
Stage II	40 (44)
Stage III	33 (37)
	
*Vascular invasion*	
Not seen	63 (70)
Intramural	11 (12)
Extramural	16 (18)

TNM=tumour node metastasis.

**Table 2:** Patient and tumour characteristics and HIF-1*α* staining

	**HIF-1*α,* number of patients (%)**		
	**Negative**	**Positive**	***P*-value**
*Gender*			*P*=0.35
Male	24 (43)	32 (57)	
Female	18 (53)	16 (47)	
*T stage*			*P*=0.48
T1/2	9 (50)	9 (50)	
T3/4	33 (46)	39 (54)	
*N stage*			*P*<0.02^*^
N0	32 (56)	25 (44)	
N1	6 (29)	15 (71)	
N2	4 (33)	8 (67)	
*Differentiation*			*P*=0.58
Well	3 (75)	1 (25)	
Moderate	33 (45)	40 (55)	
Poor	6 (46)	7 (54)	
*Dukes’ stage*			*P*=0.05
Dukes’ A	9 (53)	8 (47)	
Dukes’ B	23 (58)	17 (42)	
Dukes C1	8 (29)	20 (71)	
Dukes’ C2	2 (40)	3 (60)	
*Dukes’ stages combined*			*P*<0.02^*^
A/B	32	25	
C	10	23	
*TNM stage*			*P*<0.05^*^
Stage I	9 (53)	8 (47)	
Stage II	23 (58)	17 (42)	
Stage III	10 (30)	23 (70)	
*Vascular invasion*			*P*<0.005^*^
Absent	35 (56)	28 (44)	
Intramural	4 (36)	7 (64)	
Extramural	3 (19)	13 (81)	
*Microvessel density*^			*P*=0.32
<60 vessels	20 (53)	18 (47)	
⩾60 vessels	21 (42)	29 (58)	

HIF=hypoxia-inducible factor; TNM=tumour node metastasis.

^*^Statistically significant at 5% level.

**Table 3:** Patient and tumour characteristics and HIF-2*α* staining

	**HIF-2*α,* number of patients (%)**		
	**Negative**	**Positive**	***P*-value**
*Gender*			*P*=0.68
Male	19 (34)	37 (66)	
Female	13 (38)	21 (62)	
*T stage*			*P*=0.53
T1/2	6 (33)	12 (67)	
T3/4	26 (36)	46 (64)	
*N stage*			*P*=0.10
N0	17 (30)	40 (70)	
N1	8 (38)	13 (62)	
N2	7 (58)	5 (42)	
*Differentiation*			*P*=0.94
Well	2 (50)	2 (50)	
Moderate	25 (34)	48 (66)	
Poor	5 (39)	8 (61)	
*Dukes’ stage*			*P*=0.25
Dukes’ A	6 (35)	11 (65)	
Dukes’ B	11 (28)	29 (72)	
Dukes C1	12 (43)	16 (57)	
Dukes’ C2	3 (60)	2 (40)	
*TNM stage*			*P*=0.28
Stage I	6 (35)	11 (65)	
Stage II	11 (28)	29 (72)	
Stage III	15 (46)	18 (54)	
*Vascular invasion*			*P*=0.74
Absent	22 (35)	41 (65)	
Intramural	3 (27)	8 (73)	
Extramural	7 (44)	9 (56)	
*Microvessel density*			*P*=0.86
<60 vessels	13 (34)	25 (66)	
⩾60 vessels	18 (36)	32 (64)	

HIF=hypoxia-inducible factor; TNM=tumour node metastasis.

**Table 4:** Multivariate analysis of survival for HIF-1*α*

	**HR**	**95% CI**	***P*-value**
*HIF-1*α			
No^a^	1		
Yes	4.108	1.366-12.352	0.012
			
*Vascular invasion*			
No^a^	1		
Intramural	1.483	0.400-5.495	0.555
Extramural	2.284	0.917-5.688	0.016
			
*TNM stage*			
I^a^	1		
II	1.724	0.197-15.069	0.622
III	10.048	1.302-77.553	0.027

CI=confidence interval; HIF=hypoxia-inducible factor; HR=hazard ratio; TNM=tumour node metastasis.

a Reference values.
