# Hypoxia-inducible factor-1*α* and -2*α* are expressed in most rectal cancers but only hypoxia-inducible factor-1*α* is associated with prognosis

**DOI:** 10.1038/sj.bjc.6605242

**Published:** 2009-08-11

**Authors:** S Rasheed, A L Harris, P P Tekkis, H Turley, A Silver, P J McDonald, I C Talbot, R Glynne-Jones, J M A Northover, T Guenther

**Correction to**: *British Journal of Cancer* (2009) **100**, 1666–1673, doi: 10.1038/sj.bjc.6605026

Upon publication of this paper in Volume 100, the authors noticed an error in the results section of the Abstract, where HIF-2*α* was expressed incorrectly as HIF-1*α*. The correction is now shown below in bold.

RESULTS: MVD was higher across T- and N-stages (*P*<0.001) and associated with poor survival (Hazard ratio (HR 8.7, *P*<0.005) and decreased disease-free survival (HR=4.7, *P*<0.005). HIF-1*α* and HIF-2*α* were expressed in >50% of rectal cancers (HIF-1*α*, 54%, 48/90; HIF-2*α*, 64%, 58/90). HIF-1*α* positivity was associated with TNM stage (*P*<0.05), vascular invasion (*P*<0.005), recurrence (HR=4.5, *P*<0.005) and cancer-specific mortality (HR=5.5, *P*<0.002). **In contrast, no associations were demonstrated between HIF-2*α* and any pathological features or outcome.**

